# Liquid Biopsies from Pleural Effusions and Plasma from Patients with Malignant Pleural Mesothelioma: A Feasibility Study

**DOI:** 10.3390/cancers13102445

**Published:** 2021-05-18

**Authors:** Gabriele Moretti, Paolo Aretini, Francesca Lessi, Chiara Maria Mazzanti, Guntulu Ak, Muzaffer Metintaş, Cecilia Lando, Rosa Angela Filiberti, Marco Lucchi, Alessandra Bonotti, Rudy Foddis, Alfonso Cristaudo, Andrea Bottari, Alessandro Apollo, Marzia Del Re, Romano Danesi, Luciano Mutti, Federica Gemignani, Stefano Landi

**Affiliations:** 1 Department of Biology, Genetic Unit, University of Pisa, via Derna 1, 56126 Pisa, Italy; gabriele.moretti@student.unisi.it (G.M.); a.bottari@studenti.unipi.it (A.B.); alessandro.apollo@humanitasresearch.it (A.A.); stefano.landi@unipi.it (S.L.); 2 Fondazione Pisana per la Scienza, Via Ferruccio Giovannini 13, 56017 San Giuliano Terme, Italy; p.aretini@fpscience.it (P.A.); f.lessi@fpscience.it (F.L.); c.mazzanti@fpscience.it (C.M.M.); 3 Eskisehir Osmangazi University Lung and Pleural Cancers Research and Clinical Center, Eskisehir 26000, Turkey; guntuluak@ogu.edu.tr (G.A.); mmetintas@ogu.edu.tr (M.M.); 4 Department of Chest Diseases, Medical Faculty, Eskisehir Osmangazi University, Eskisehir 26000, Turkey; 5 IRCCS Ospedale Policlinico San Martino, Clinical Epidemiology, 16132 Genova, Italy; ceciliafrancesca.lando@hsanmartino.it (C.L.); rosa.filiberti@hsanmartino.it (R.A.F.); 6 Division of Thoracic Surgery, Cardiac-Thoracic and Vascular Department, University Hospital of Pisa, 56124 Pisa, Italy; m.lucchi@med.unipi.it; 7 Preventive and Occupational Medicine, University Hospital of Pisa, 56126 Pisa, Italy; abonotti@yahoo.it; 8 Department of Translational Research and of New Technologies in Medicine and Surgery, University of Pisa, 56126 Pisa, Italy; rudy.foddis@med.unipi.it (R.F.); alfonso.cristaudo@med.unipi.it (A.C.); 9 Division of Pharmacology, Department of Internal Medicine, University of Pisa, 55, Via Roma, 56126 Pisa, Italy; marzia.delre@ao-pisa.toscana.it (M.D.R.); romano.danesi@unipi.it (R.D.); 10 Sbarro Institute for Cancer Research and Molecular Medicine, Center for Biotechnology, College of Science and Technology, Temple University, Philadelphia, PA 19104, USA

**Keywords:** malignant pleural mesothelioma, liquid biopsies, circulating tumor DNA, plasma, cancer-specific mutations, genomics, cancer biomarkers

## Abstract

**Simple Summary:**

Patients with malignant pleural mesothelioma (MPM) often have to wait a long time before receiving a diagnosis. To contribute to the research on this neoplasm, we analyzed various samples of tumor biopsy and the relative liquid biopsies from both plasma and pleural fluid. We tested the possibility of obtaining information about the tumor in a quicker and less invasive way compared to the usual solid biopsy. We performed NGS on blood and tumor samples from patients and obtained a list of somatic mutations. With the digital droplet PCR technique, we tested the respective pleural fluids and plasma for the previously found mutations. We discovered that pleural fluid is a good proxy to obtain the mutational landscape of the MPM. We also tracked tumor DNA in plasma, leading to the idea that this could be used in a clinical setting to perform follow-ups of patients and monitor drug responses.

**Abstract:**

Background: Malignant pleural mesothelioma (MPM) is a fatal tumor with a poor prognosis. The recent developments of liquid biopsies could provide novel diagnostic and prognostic tools in oncology. However, there is limited information about the feasibility of this technique for MPMs. Here, we investigate whether cancer-specific DNA sequences can be detected in pleural fluids and plasma of MPM patients as free circulating tumor DNA (ctDNA). Methods: We performed whole-exome sequencing on 14 tumor biopsies from 14 patients, and we analyzed 20 patient-specific somatic mutations with digital droplet PCR (ddPCR) in pleural fluids and plasma, using them as cancer-specific tumor biomarkers. Results: Most of the selected mutations could be detected in pleural fluids (94%) and, noteworthy, in plasma (83%) with the use of ddPCR. Pleural fluids showed similar levels of somatically mutated ctDNA (median = 12.75%, average = 16.3%, standard deviation = 12.3) as those detected in solid biopsies (median = 21.95%; average = 22.21%; standard deviation = 9.57), and their paired difference was weakly statistically significant (*p* = 0.048). On the other hand, the paired difference between solid biopsies and ctDNA from plasma (median = 0.29%, average = 0.89%, standard deviation = 1.40) was highly statistically significant (*p* = 2.5 × 10^−7^), corresponding to the important drop of circulating somatically mutated DNA in the bloodstream. However, despite the tiny amount of ctDNA in plasma, varying from 5.57% down to 0.14%, the mutations were detectable at rates similar to those possible for other tumors. Conclusions: We found robust evidence that mutated DNA is spilled from MPMs, mostly into pleural fluids, proving the concept that liquid biopsies are feasible for MPM patients.

## 1. Introduction

Malignant pleural mesothelioma (MPM) is a fatal cancer that arises from the mesothelial cells of the pleura. Asbestos exposure and the host’s predisposing conditions (e.g., inherited mutations within *BAP1* or a chronic inflammatory state of the pleura [1]) play a role in the carcinogenesis of this neoplasm. Fibers are hypothesized to trigger a chronic inflammatory status, inducing a condition known as “frustrated apoptosis” of macrophages [2] and leading to increased production of oxygen reactive species, DNA damage, and cell proliferation, eventually initiating and promoting the malignant process [3,4]. The latency between exposure to asbestos fibers and diagnosis usually takes decades [5], and the first symptoms (which include, but are not limited to, chest pain, breathing difficulties, dyspnea, or increased abdominal volume) are common to other respiratory conditions [6], making a prompt diagnosis very difficult. Widely used imaging methods are not sufficient for the diagnosis of MPM. Thus, to achieve a reliable diagnosis, one needs to perform a biopsy through video-assisted thoracoscopy (VATS) [7,8], although this invasive procedure cannot be routinely used to assess the successive genetic changes.

Liquid biopsies (LBs) represent an innovative approach under development and consist of the analysis of genetic material extracted from body fluids. Events like apoptosis, necroptosis, and cell migration may result in the dispersion of tumor cells or their debris in the fluids surrounding the tumor mass [9]. Therefore, under these circumstances, it is possible to detect circulating tumor cells (CTCs), circulating cell-free tumor DNA (ctDNA), tumor proteins, and tumor-derived extracellular vesicles (tEVs, which include exosomes) in plasma, urine, or other body fluids [9]. Numerous studies have confirmed the possibility of gaining information on many kinds of tumors via blood samples. At first, CTCs were isolated and examined to get more insight into tumor progression and mutational history [10]. CTC phenotypic characterization and count can give hints on the tumor stage and expansion, whereas their DNA can provide information about the tumor mutational landscape [10]. Similarly, ctDNA could also be useful for LBs. In cancer patients, up to 1% of circulating nucleic acids are derived from tumor cells. The ability to isolate and analyze this DNA has made it possible to detect circulating mutations deriving from hepatocellular, breast, lung, and pancreatic carcinoma [11,12,13,14]. Evidence suggests the possibility of inferring or confirming the diagnosis of these tumors and performing clinical follow-ups by tracking the mutational load in response to therapies. In specific cases, such as lung adenocarcinoma, the monitoring of mutated ctDNA could provide important information to adjust a personalized therapy based on the use of anti-EGFR drugs [15,16]. On the other hand, other tumors (such as glioblastoma) are not equally capable of spilling ctDNA into the bloodstream [17], and the knowledge, in this regard, on MPM is limited. CtDNA from MPM patients has been analyzed in two previous studies. In 2012, higher DNA integrity was detected in cytologically negative pleural fluids (PFs) from 16 MPM patients (median = 1.2) compared to 23 noncancer patients (median = 0.8). The conclusion is that this biomarker, along with others (e.g., mesothelin), could improve the specificity and sensitivity needed to discriminate MPM from non-MPM patients [18]. More recently, in 2018, 10 MPM patients were analyzed for ctDNA (half of them were treatment-naïve). In this work, tumor biopsies were sequenced, and ctDNA was investigated in plasma samples via digital-droplets PCR (ddPCR). The authors showed that more than half of the treatment-naïve subjects showed positive droplets for mutated ctDNA in their blood samples, demonstrating the presence of tumor-specific mutations in circulating DNA [19]. However, the number of analyzed patients was limited, and not all of them showed mutated ctDNA from MPM in their bloodstream. Furthermore, no other fluids have been analyzed in the attempt to find an alternative approach to increasing the analysis’ sensitivity. In order to fill the lack of knowledge on this topic, we analyzed a series of 14 MPM patients and carried out more systematic research on solid tumor biopsies, PF, and plasma withdrawn from the same patient. Thus, we could show that the share of somatically mutated cancer-specific DNA from PFs is similar to that detected in solid biopsies and that the same somatic mutations can also be detected, in tiny amounts, in the plasma of the same patient. Therefore, this feasibility study provides evidence that, in the future, PFs and plasma could constitute a valuable source of information, allowing for the diagnosis, follow-up, and stratification of MPM patients.

## 2. Materials and Methods

### 2.1. Patients Cohorts

We analyzed samples from patients diagnosed with MPM from three different hospital centers; we divided them into two groups based on the availability of blood samples.

Group GE consisted of 7 frozen tumor biopsies from San Martino Hospital in Genoa (Italy); each of them was associated with frozen samples of plasma and PF. Biopsies were about 1 mm^3^ in size. PF, collected from patients’ pleural effusions, and plasma samples were available in different amounts for each subject, ranging from 3 to 6 mL. Patients were diagnosed with MPM at an average age of 71 during the period 2002–2012. All of them were deceased at the moment of this analysis, having a median survival time since diagnosis of 4.6 months, with a minimum of 1 month and a maximum of 33.

Group PT (Pisa and Turkey) consisted of biopsies, frozen whole-blood, and frozen plasma samples from 2 patients (P) from the University Hospital of Cisanello in Pisa (Italy) and 5 patients (T) from Eskişehir Osmangazi University Hospital in Turkey in the period 2017–2019. Patients were diagnosed at a median age of 68. All of them were deceased at the time of the analysis, with a median survival time since diagnosis of 8.9 months and a minimum of 6.4 months and a maximum of 15.5 months. The size of the biopsies was about 1 mm^3^, and the volumes of whole blood and plasma were 2 and 1.5 mL, respectively. For patients T, a sample of PF was also available (2 mL). Patients’ information is reported in Table S1.

### 2.2. Sequencing and Filtering

In order to discern somatic from germline mutations, whole-exome sequencing (WES) was carried out on solid biopsies and buffy coats withdrawn from the same patient of Group PT, while specific algorithms and filtering procedures were employed for the patients of Group GE. Genomic DNA was extracted using a PureLink™ Genomic DNA Mini Kit (Thermo Fisher Scientific; Waltham, MA, USA), following manufacturer protocol. This was used for both blood and tumor samples. The final DNA samples’ concentration was measured with a Qbit3 (Thermo Fischer Scientific; Waltham, MA, USA). WES was performed on a NextSeq 550 (Illumina; San Diego, CA, USA) and the library was prepared using the kit from the same producer (Nextera DNA Flex Pre-Enrichment Library Prep and Enrichment). Sequencing indexes were also provided by the same manufacturer. Alignment of the resulting FASTA files was performed with Burrows-Wheeler Aligner software [20]. The calling of somatic mutations for the tumor samples was performed with VarScan [21], where paired blood was available; for the remaining cases, GATK tool Mutect2 [22] was used. The resulting single nucleotide variations (SNVs) were annotated using the VEP online tool from the Ensembl portal (http://grch37.ensembl.org/Homo_sapiens/Tools/VEP/ accessed on 16 March 2019).

Since no blood was available for Group GE whereas it was available for the PT group, two alternative filtering procedures, FGE and FPT, were carried out.

FGE was carried out as follows. To maximize the chances of selecting a somatic mutation, we considered those with a ratio of alternative allele reads (i.e., alternative depth, AD) to total reads (i.e., total depth, TD) of 0.25 or lower. This is because such a ratio may originate from mutated tumor cells, whereas a higher ratio could indicate a homozygous or heterozygous mutation present in all the sample’s cells, which is less likely somatic. Another parameter of FGE filtering excluded the mutations within noncoding regions. This was done to allow an easier interpretation of the functional consequence of the variation in the context of the carcinogenic progression. The last filter condition required mutations to have an AD greater than 20X and a minor allele frequency (MAF) in the population ≤10^−4^, according to gnomAD (https://gnomad.broadinstitute.org accessed on 16 March 2019). The former parameter ensures a good NGS quality, while the latter allows us to take into account the negative selection a mutation undergoes in the population, decreasing the possibility of it being germline.

FPT consisted, firstly, in the use of VarScan2, a software based on the statistical analysis of a coverage value for both reference and alternative bases, comparing that found in blood with that of the paired tumor. Then, further filtering was applied using the following criteria: (i) a minor allele frequency (MAF) <1% among Europeans, according to gnomAD, (ii) a read depth ≥20X, and (iii) AD = 0 in the blood sample.

From the final list of SNVs obtained with the filtering procedures, up to 5 mutations per patient were selected for further experimental validation. This last choice did not follow a strict criterion but was based on a variety of criteria that included (i) mutations present on a gene already filed for MPM within COSMIC or TCGA databases (https://cancer.sanger.ac.uk/cosmic, https://www.cancer.gov/tcga accessed on 16 March 2019), (ii) the lack of any repeated sequence in the neighboring region of the SNV, and (iii) the lack of paralogues/gene families of the mutated genes.

### 2.3. Validation and Biostatistical Analysis

SNVs selected following WES were verified in tumor biopsies with an allele-specific oligonucleotide and a real-time quantitative PCR method (ASO–qPCR). For each SNV, the real-time curve obtained with mutation-specific primers was compared with the curve obtained with specific primers designed for the wild-type allele. The results were also compared to the same assay performed on DNA extracted from the whole blood of a healthy subject (reference). This analysis is not quantitative enough to measure the amount of mutated DNA. On the other hand, it is inexpensive and sensitive enough to verify the presence/absence of small amounts of mutated alleles among a plethora of wild-type alleles. Experiments were performed with a CFX96 thermal cycler (Bio-Rad; Hercules, CA, USA) using 5× HOT FIREPol^®^ EvaGreen^®^ qPCR Mix Plus (Solis Biodyne; Tartu, EE, Estonia) and custom oligonucleotides primers (Europhins Genomics, Louxemburg, LU). When ASO–qPCR confirmed the mutation in tumor DNA, we proceeded by using the more sensitive ddPCR for quantification. Thus, for each patient, ddPCR was applied on tumor samples as well as other available fluids.

Circulating DNA was extracted using a QIAamp Circulating Nucleic Acid Kit (Qiagen; Venlo, NL, Netherlands), and the concentrations were measured with a Qbit (Thermo Fisher Scientific; Waltham, MA, USA). DdPCR was used to measure and compare the amount of mutated DNA (using mutation-specific probes) in tumor, blood, PF, and plasma. DNA from a healthy individual and a blank (buffer only) were used as negative controls. We used a QX100 droplet generator to form the reaction droplets and a QX200 droplet reader (Bio-Rad; Hercules, CA, USA) to get the results. The PCR amplification reaction was performed in a T100 thermal cycler from the same manufacturer. For each SNV, we used a pair of TaqMan-like probes, each targeted either to the variant or the common allele, the former being labeled with FAM and the latter with HEX fluorophore. Probes and primers were designed using Bio-Rad’s online probes design tool. The reaction mix used was Bio-Rad’s ddPCR Supermix for Probes (No dUTP). Differences in the amount of mutated ctDNA from plasma or PF compared to that measured in solid biopsies (as reference) were evaluated with a paired Student’s *t*-test analysis, following arcsin transformation for non-normally distributed data, and the nonparametric Wilcoxon signed-rank test.

## 3. Results

### 3.1. GE patients, NGS Analysis

For Group GE, NGS analysis yielded an average of 78.67 million reads per tumor, with a mean length of 122 bases. Across all samples, 87.1% of the reads were correctly aligned with the reference, with a mean mapping quality of 59.5 and an average coverage inside the exome regions of 97.7X. After the analysis with the Mutect2 tool, which computed all mismatches in the reads to find mutations, we obtained 97,826 to 123,405 variants, depending on the sample (Table 1). Of those variants, 6.9–10.8% were indels (insertion/deletions) and 89.2–93.1% were SNVs, with an average median coverage of 102X. The values of this parameter fitted a Laplace distribution with a mean of 41.5X.

Then, FGE was optimized to maximize the likelihood of selecting truly somatic mutations. Firstly, variants with the number of AD reads (the ones covering the alternative allele) lower than 25% (arbitrarily chosen), relative to TD (total depth), were positively selected. Between 1065 and 3053 variants passed this step, depending on the sample (Table 1). The second step of selection included only the SNVs within the coding regions (between 593 and 1342). A further step of the FGE procedure was carried out by excluding the variants with less than 20 reads covering the genomic position. Then, among the selected SNVs, those with a MAF ≥ 10^−4^ (global, according to gnomAD, arbitrary threshold) were excluded as well. A number of SNVs, between 42 and 184, remained in the final list.

### 3.2. In GE Patients, Selected Somatic Mutations Were Detected in ctDNA from Plasma and PFs

A total of 25 mutations within 25 genes in 7 subjects were evaluated with ASO–qPCR in tumor biopsies, and 14 were confirmed by this method. The list encompassed *COL1A2*, *BACE2*, *MYBPC1*, *TRPC7*, *ARPP21*, *OR4K2*, *HIST1H2AD*, *OR5AC2*, *SZT2*, *AMPH*, *SPTAN1*, *NLGN1*, *DICER1*, and *FLI1*; most of them had already been reported as somatically mutated in the MPM patients, according to COSMIC or TCGA databases (Table 1). Four SNVs within *BAP1, LATS2, MUC16,* and *FLG*, the genes most frequently mutated in MPM, together with other 7 mutations in *POTEF*, *RAD50*, *FGFR1*, *UNC79*, *ERBB4*, *CSMD3*, and *CCNL2*, could not be confirmed by ASO–qPCR and were not investigated further with ddPCR.

The measurements carried out with ddPCR on the 14 confirmed mutations showed that 3 (*COL1A2*-rs773494330, ID = 696; *SZT2*-rs760370909, ID = 2324; *DICER1*-rs775912475, ID = 2829) could not be detected in tumor biopsies with ddPCR, whereas positive results were obtained for 11 mutations found in the biopsies of 7 patients (3 patients were positive for 1 mutation and 4 patients were positive for 2 mutations), as reported in Table 1.

When PFs were analyzed, samples from 6 patients were available. One, ID = 696, could not be analyzed because the amplification failed several times, even after adopting alternative protocols for DNA extraction, suggesting the presence of unknown PCR inhibitors. Thus, only 10 mutations could be compared between tumor biopsies and PFs. Interestingly, for 7 of them, the share of mutated alleles measured in PFs was similar (ranging from 10.24% to 20.20%) to that measured in the respective tumor biopsies. The remaining three mutations showed a reduced amount; however, they were still detectable to a significant extent: *TRPC7*-rs566980923 (ID = 1148), 12.5% in tumor and 4.9% in PF; *AMPH*-COSM1673120 (ID = 2324), 15.1% (tumor) and 4.01% (PF); *NLGN1*-COSM479730 (ID = 2438), 21.95% and 1.59%, respectively (Table 1).

Interestingly, 9 out of 11 mutations of tumor biopsies were also detected in the ctDNA from plasma. Two, *TRPC7*-rs566980923 ID = 1148 and *NLGN1*-COSM479730 ID = 2438, were undetected, and this was in agreement with the low quantity already detected in the respective PF samples. Of the 9 detectable mutations, 2 (*MYBPC1*-rs752347381 ID = 1148 and *OR4K2*-rs757533510 ID = 2294) were likely germline. In fact, for these mutations, the percentage of the alternative allele in PF and plasma was about 25% and of a similar range to that measured in the tumor biopsies. However, as reported in Table 1, the remaining seven mutations were most likely somatic and showed a percentage ranging between 0.16% and 0.79%, whereas their corresponding share within the tumor biopsies ranged between 11.1% and 23.05%. The one showing the highest amount was *OR5AC2*-rs1021819573 (ID = 2294) with a percentage of 5.57 (it was 11.1 in the tumor). Thus, 6 out of 7 patients showed ctDNA in their plasma. Only patient ID = 1148 could not be traced using the selected mutations. Unfortunately, the analysis of the other patient’s mutations could not be carried out because of the lack of additional vials of plasma.

### 3.3. PT Patients, NGS Analysis

NGS analysis on Group PT yielded an average of 80.66 million reads for each subject’s tumor sample. The average read length was 98 bases. Across all samples, 70% of the reads were correctly aligned in the exome reference region, with a mapping quality of 54.9 and an average coverage of 72X. After the analysis with the software VarScan2 for each tumor–blood pair, we identified between 102,753 and 130,073 SNVs, of which 1948–3120 were marked as somatic (Table 2). The TD values had a median of 45 and fitted a Laplace distribution with a mean of 50X. The indels were not evaluated in our assays; however, they consisted of a share ranging from 8.89% to 14% of the total variations. FPT consisted of selecting mutations within coding regions, eliciting from 158 to 281 SNVs. Then, SNVs with TD < 20 and a population MAF > 1% (global according to gnomAD) were excluded. The resulting 54–104 SNVs were further filtered by including only mutations with AD = 0 in blood samples, yielding 2 to 42 mutations. Finally, among the available variants filtered for both groups, we selected two mutations per sample for further analyses, as specified in “Materials and Methods” (Table 1 and Table 2).

### 3.4. Selected Mutations for Group PT were also Detected in the ctDNA from Plasma and PF

Fourteen mutations were analyzed with ASO–qPCR in Group PT, and twelve (two for each T patient and one for each P patient) could be confirmed in the tumor biopsies (*OR10A4*-rs547489107 and *NLRP6*-NM_138329.2:c.403G > T were undetected), as reported in Table 2. Thus, we used ddPCR to measure the amount of mutated DNA within the tumor biopsies, and only one mutation (*SS18*-NM_001007559.3:c.98A > G, of subject 01T) could not be detected. Of the remaining 11 mutations in 10 genes (*BAP1* occurred twice), we found that *BAP1*, *NF2*, *FAT1*, *JADE1*, and *FLT1* were already present in the COSMIC and TCGA databases for MPM patients. For eight variants, the percentage of mutated DNA analyzed was of a similar extent to that yielded by NGS, considering an expected 10% error rate. On the other hand, *JADE1*- rs775483821 (ID = 01T) had 7.48% of mutated DNA in ddPCR opposed to 24.53% of the NGS, whereas *BAP1*-COSM4411449(C > T) (ID = 02T) had 33.50% vs. 21.88% and *NF2*- NM_016418.5:c.985A > T (ID = 03P) showed 15.85% vs. 27.03%, respectively. In PF, among the 11 mutations detected in tumor biopsies, 2 (*VIL1*- NM_007127.3:c.2070C > T and *NF2*-NM_016418.5:c.985A > T) could not be analyzed due to the lack of biological specimens of subjects 02P and 03P, whereas 1 (*FAT1*- rs776531396; ID = 05T) was undetectable. The remaining 8 SNVs were detected with percentages compatible with those observed in tumor biopsies, ranging from 12.75% to 39.70%. The only exception was patient 04T, whose mutations (*FAM71B*- rs1404037352 and *CSMD2*- rs770364421) had lower mutated allele relative abundance in PF compared with the tumor sample, namely, 9.7% against 30.80% and 8.95% against 25%, respectively.

The 11 mutations were also investigated in plasma samples. For patient 04T, we could not assay two mutations because of the lack of biological specimens. Of the remaining nine mutations, eight were also detectable in the patients’ plasma, whereas *FAT1*- rs776531396 (ID = 05T) was undetectable, in agreement with the lack of detection in his PF. In plasma, the eight mutations could be detected, with percentages ranging from 0.14% to 2.68%. All these results are summarized in Table 2.

Considering both groups of patients and excluding the two mutations highly suspected to be of germline origin and the one not detected in solid biopsy, the percentages of mutated DNA detected in solid biopsies were higher than those detected in ctDNA from PFs: median = 21.95 vs. 12.75 (respectively); average ± st.dev = 22.21 ± 9.57 vs. 16.3 ± 12.3. This difference was statistically significant (*p* = 0.0237) when analyzed with Student’s *t*-test for paired data and not statistically significant when analyzed with nonparametric Wilcoxon’s test (*p* = 0.0648). The difference was much greater when compared to ctDNA from plasma (median = 0.29, average 0.89 ± 1.40), providing a high statistical significance to the same statistical tests (*p* = 2.49 × 10^−7^ and *p* = 3.2 × 10^−4^, respectively).

## 4. Discussion

In this study, we report a positive feasibility study that in MPM patients, ctDNA is present in PF at high concentrations and cancer-specific DNA can be detected in plasma, although at low percentages. Therefore, we provide evidence that LBs for patients with MPM is feasible, and this could represent a potentially important tool for the diagnosis, therapy, follow-up, and stratification of patients, especially with pleural effusions.

It is noteworthy that we ruled out the possibility of selected germline mutations, either by using stringent filtering procedures or by carrying out WES of the buffy coat, when available. Thus, the present data reinforce and extend the preliminary evidence reported by Hylebos et al. [19], where only 3 out of 10 patients presented ctDNA in plasma samples. In that study, only one mutation was assayed, and no PFs were available. In our study, we started from a selection of 39 total mutations, and 22 could be confirmed in solid biopsies, allowing further investigations in PFs and plasma. Since we considered these SNVs enough for our purposes, we did not pay further attention to the remaining 17 mutations. Likely, they could not be validated because of poor ASO–qPCR probe performance.

**Figure 1:**
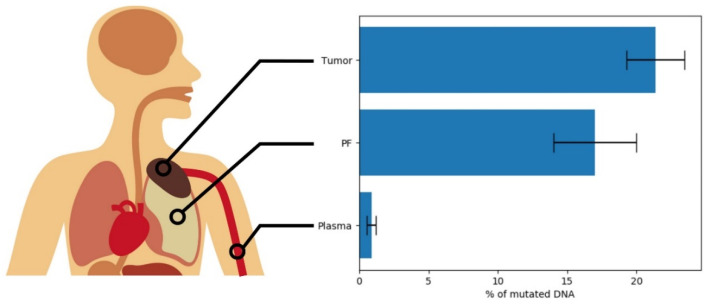
Mean levels of mutated DNA found in the samples from three sources: solid tumor, pleural fluid, and plasma.

In two GE patients, two mutations showed high and similar percentages in tumor, PF, and plasma, strongly suggesting a germline origin. This result was not surprising because, despite the stringent filtering procedure we applied, GE patients’ buffy coat was lacking, and WES could not be carried out. However, the remaining 20 were enough for investigating whether MPM patients could carry ctDNA in PF or plasma. With the exception of subjects 696 (PCR could not work for an inhibitor), 02P, and 03P (PFs not available), 16 out of 17 mutations could be detected in PF. The percentages of the mutated allele detected differed by about 7.5%, on average, from those found in the tumor biopsies, a value falling within the intrinsic error of NGS technology (Figure 1). This fact indicates that DNA extracted from PF is a good proxy for its counterpart obtained from the solid tumor. In the future, DNA from PF could be employed instead of the classical solid biopsies to gain insights on the cancer’s mutational landscape with much less distress for the patient. Moreover, 15 out of 18 analyzable mutations were also detectable in plasma, with relative abundances varying from 0.14% to 5.57%. Since only a few milliliters of plasma were available from the biobank, we could not analyze a high number of DNA copies in plasma. It is conceivable that the analysis of higher amounts of DNA could have elicited positive results in the three negative cases as well.

The fact that MPM is a locally spreading tumor on the pleural surface could provide a good explanation of the high amount of ctDNA detected in PFs and the low amount detected in plasma. We can hypothesize that the observed interindividual variability of ctDNA levels could be ascribed to the relative amounts of subclones tracked with the picked mutation, the aggressiveness of the subclone carrying the picked mutation, or to the mechanisms involved in the release of tumor DNA.

We foresee that the use of PF or plasma could be very important in the diagnosis process and for a noninvasive clinical follow-up of the patients. An earlier diagnosis could be carried out by integrating the results of ctDNA analysis with currently available biomarkers, such as serum soluble mesothelin levels, and other epigenetic biomarkers under research, such as the expression of the circulating microRNAs miR-16, miR-17, miR-126, miR-486 or CpG methylation at *CDKN2A* or *SFRP* genes [23]. In fact, once the tumor is characterized for its genetic background, specific mutations could be used to monitor the evolution of the disease, allowing early detection of its worsening before any clinical observation. The analysis of cancer-specific mutations through the use of LBs could also allow more accurate monitoring of responses to therapies. With our work, we enlighten the versatility of this method to obtain genetic information on MPM using PF and plasma as starting materials.

One limitation to the present study consisted of the limited clinical information available from the biobanks of the samples G and P. It could be hypothesized that the percentage of mutated copies would be higher in patients presenting the tumors at advanced stages, conceivably with the idea of a higher extent of ctDNA released from largely spread tumors or metastases. At the present time, it is not possible to know whether the mutated DNA could also be detected in LBs from MPM patients with earlier stages of the disease. Future research should be encouraged to approach this task. However, we analyzed whether the amount of mutated DNA could correlate with patients’ overall survival (a proxy of the tumor staging), and we could not find any statistically significant correlation. We could hypothesize that this is due to the low statistical power for this type of analysis or to the fact that all MPM patients are diagnosed at late stages. Given the possibility of gathering more clinical and histological data about the tumor, such as cell type, tumor stage, and treatment response, our results may prove even more useful in the clinical field.

## 5. Conclusions

In summary, this study showed that LBs are feasible in MPM, paving the way for novel tools in the clinical management of these patients. It has been figured out that once the profile of MPM’s somatic mutations is fully achieved, the choice of the therapy, its effectiveness, and/or the occurrence of relapses can also be monitored by using PF and plasma as a source of ctDNA.

## Figures and Tables

**Table 1:** Selected mutations analyzed for Group GE after FGE filtering and relative ASO–qPCR and ddPCR results.

ID	NGS											ddPCR		
							Selected SNVs							
									Reads			% of Variant Allele		
	SNV Tot	AD/TD ≤ 0.25	Coding	MAF < 10^−4^ & AD > 20	Gene	Type	ID or Position	MAF ^a^	AD/TD	AD (%)	qPCR	Tumor	PF	Plasma
696	104,367	1330	797	45	*POTEF*	S	NM_001099771.2:c.2118T > C	NA	22/112	19.64	No	-	-	-
					*COL1A2*	S	rs773494330	4.00 × 10^−6^	23/110	20.91	Yes	0.00	Inhibitor	0.00
					*BACE2*	M	rs770736773, COSM5907863	4.00 × 10^−5^	20/91	21.98	Yes	23.05	Inhibitor	0.16
1148	106,264	1985	938	122	*BAP1*	FS	NM_004656.1:g.52443623del	NA	31/219	14.16	No	-	-	-
					*MUC16*	M	rs75266616	9.11 × 10^−5^	22/100	22.00	No	-	-	-
					*RAD50*	FS	rs772667708, COSM1433045	2.10 × 10^−4^	23/197	11.68	No	-	-	-
					*MYBPC1*	M	rs752347381	8.00 × 10^−6^	26/140	18.57	Yes	20.80	23.80	26.55
					*TRPC7*	M	rs566980923	<10^−6 b^	40/256	15.63	Yes	12.50	4.90	0.00
1725	98,442	1981	920	88	*ARPP21*	M	rs1481888266	8.88 × 10^−6^	22/124	17.74	Yes	16.65	16.00	0.26
					*OR4K2*	M	rs757533510	4.00 × 10^−6^	25/98	25.51	Yes	22.40	22.80	24.05
2294	101,976	1810	886	96	*FLG*	S	rs564106508, COSM5531298	3.60 × 10^−5^	24/196	12.24	No	-	-	-
					*FGFR1*	IF	rs138489552	7.20 × 10^−5^	22/143	15.38	No	-	-	-
					*UNC79*	M	NM_020818.1:g.94110000C > A	NA	21/99	21.21	No	-	-	-
					*HIST1H2AD*	M	NM_021065.1:g.26199201G > A	NA	24/217	11.06	Yes	12.05	16.45	0.79
					*OR5AC2*	S	rs1021819573	2.72 × 10^−5^	25/163	15.34	Yes	11.10	12.10	5.57
2324	123,405	2852	1178	184	*ERBB4*	M	NC_000002.12:g.211561993C > T	NA	21/173	12.14	No			
					*SZT2*	M	rs760370909	4.00 × 10^−6^	27/143	18.88	Yes	0.00	0.00	0.00
					*AMPH*	M	COSM1673120 (C > A)	NA ^c^	25/171	14.62	Yes	15.10	4.01	0.17
2438	97,826	1065	593	42	*SPTAN1*	M	NM_001130438.3:c.252G > C	NA	28/141	19.86	Yes	21.85	20.20	0.52
					*NLGN1*	M	COSM479730 (G > T)	NA ^d^	23/104	22.12	Yes	21.95	1.59	0.00
2829	105,292	3053	1342	116	*LATS2*	S	NM_014572.3:c.1698C > A	NA	22/173	12.72	No	-	-	-
					*CSMD3*	M	COSM6112252 (G > T)	NA ^e^	47/222	21.17	No	-	-	-
					*CCNL2*	M	NM_030937.3:c.1322747G > T	NA	23/145	15.86	No	-	-	-
					*DICER1*	M	rs775912475	8.00 × 10^−6^	22/234	9.40	Yes	0.00	0.00	0.00
					*FLI1*	M	rs1288594591	4.00 × 10^−6^	25/156	16.03	Yes	11.88	10.24	0.55

MAF = minor allele frequency; TD = total depth; AD = alternative depth, MA = mutated allele; PF = pleural fluid; S = synonymous; M = missense; FS = frame shift; IF = in frame; SG = stop gain. ^a^ According to gnomAD (https://gnomad.broadinstitute.org, accessed on 16 March 2019), global frequency. ^b^ This SNV does not have a frequency in gnomAD (https://gnomad.broadinstitute.org accessed on 16 March 2019). ^c^ There is a nearby SNP, rs140004238 (G > A), with a global frequency of 3.98 × 10^−6^, at 7:38516516 (+1bp). ^d^ There is a SNP, rs1349519137 (G > C), with a global frequency of 3.19 × 10^−5^, at the same genomic position. ^e^ There is a nearby SNP, rs1377012777 (A > G), with a global frequency of 3.98 × 10^−6^, at 8:113988191 (+2 bp).

**Table 2:** Selected mutations analyzed for Group PT after FPT filtering and relative ASO–qPCR and ddPCR results.

ID	NGS													ddPCR			
								Selected SNVs									
										Reads				% of Variant Allele			
	SNV Tot	Somatic ^¥^	Coding	MAF < 1% & TD > 20	AD = 0 in Blood	Gene	Type	ID or Position	MAF ^a^	AD/TD (Blood)	AD/TD (Tumor)	AD (%) (Tumor)	qPCR	Blood	Tumor	PF	Plasma
01T	122,995	2509	158	54	2	*JADE1*	S	rs775483821	3.99 × 10^−6^	0/81	13/53	24.53	Yes	0.00	7.48	12.75	0.20
						*SS18*	M	NM_001007559.3:c.98A > G	NA	0/44	6/20	30.00	Yes	0.06	0.00	0.10	-
02T	102,753	1948	203	97	22	*FLT1*	M	NM_002019.4:c.3697C > A	NA	0/125	23/55	41.82	Yes	0.07	32.85	24.85	2.68
						*BAP1*	SG	COSM4411449(C > T)	NA ^b^	0/145	21/96	21.88	Yes	0.00	33.50	22.90	1.39
03T	117,237	2525	281	104	39	*DCAF8*	M	COSM319811	NA	0/105	47/129	36.43	Yes	0.00	33.95	36.35	0.58
						*PEG10*	M	rs368939059 COSM1093296	8.03 × 10^−6^	0/78	42/102	41.18	Yes	0.00	35.90	39.70	1.65
04T	130,073	2755	152	75	27	*FAM71B*	M	rs1404037352	1.6 × 10^−5^	0/145	38/138	27.54	Yes	0.00	30.80	9.70	N.A.
						*CSMD2*	S	rs770364421, COSM5951197	6.60 × 10^−5^	0/118	32/117	27.35	Yes	1.45	25.00	8.95	N.A.
05T	126,782	2833	227	81	23	*FAT1*	M	rs776531396	4.01 × 10^−6^	0/150	61/236	25.85	Yes	0.07	23.25	0.00	0.00
						*BAP1*	SG	rs771713346, COSM6945226	4.00 × 10^−6^	0/93	18/75	24.00	Yes	0.00	31.75	36.50	0.14
02P	128,899	3027	266	66	13	*VIL1*	S	NM_007127.3:c.2070C > T	NA	0/293	29/143	20.28	Yes	0.04	13.20	N.A.	0.29
						*OR10A4*	M	rs547489107	4.40 × 10^−5^	0/137	14/62	22.58	No	-	-	N.A.	-
03P	122,214	3120	239	88	42	*NF2*	SG	NM_016418.5:c.985A > T	NA	0/155	10/37	27.03	Yes	0.00	15.85	N.A.	0.14
						*NLRP6*	SG	NM_138329.2:c.403G > T	NA	0/123	30/151	19.87	No	-	-	N.A.	-

MAF = minor allele frequency; TD = total depth; AD = alternative depth; PF = pleural fluid; S = synonymous; M = missense; SG = stop gain. ^¥^ Predicted by VarScan2 tool (DOI:10.1101/gr.129684.111); ^a^ according to gnomAD (https://gnomad.broadinstitute.org accessed on 16 March 2019), global frequency. ^b^ There is a SNP, rs770127999 (C > A), with a global frequency of 4.00 × 10^−6^, at the same genomic position.

## Data Availability

Data is contained within the article or supplementary materia.

## Supplementary Materials

The following are available online at https://www.mdpi.com/article/10.3390/cancers13102445/s1, Table S1: patients clinical data.

## Abbreviations

AD	number of the reads of the alternative allele (i.e.,: alternative depth)
ASO–qPCR	allele-specific oligonucleotide and real-time quantitative PCR
CTCs	circulating tumor cells
ctDNA	circulating cell-free tumor DNA
ddPCR	digital-droplets PCR
FGE	filtering WES data in GE patients
FPT	filtering of WES data in PT patients
GE	patients from Genova
LB	liquid biopsies
MAF	minor allele frequency
MPM	malignant pleural mesothelioma
NGS	next-generation sequencing
P	patients from Pisa
PFs	pleural fluids
PT	patients from Pisa and Turkey
SNVs	simple nucleotide variants
T	patients from Turkey
TD	total number of reads (i.e., total depth)
tEV	tumor-derived extracellular vesicles
VATS	video-assisted thoracoscopy
WES	whole-exome sequencing.
